# The social life of Norway rats (*Rattus norvegicus*)

**DOI:** 10.7554/eLife.54020

**Published:** 2020-04-09

**Authors:** Manon K Schweinfurth

**Affiliations:** School of Psychology and Neuroscience, University of St AndrewsSt AndrewsUnited Kingdom; Weizmann Institute of ScienceIsrael; Harvard UniversityUnited States

**Keywords:** rattus norvegicus, sociality, behaviour, cognition, model organism, welfare

## Abstract

The Norway rat has important impacts on our life. They are amongst the most used research subjects, resulting in ground-breaking advances. At the same time, wild rats live in close association with us, leading to various adverse interactions. In face of this relevance, it is surprising how little is known about their natural behaviour. While recent laboratory studies revealed their complex social skills, little is known about their social behaviour in the wild. An integration of these different scientific approaches is crucial to understand their social life, which will enable us to design more valid research paradigms, develop more effective management strategies, and to provide better welfare standards. Hence, I first summarise the literature on their natural social behaviour. Second, I provide an overview of recent developments concerning their social cognition. Third, I illustrate why an integration of these areas would be beneficial to optimise our interactions with them.

## Introduction

The Norway rat (*Rattus norvegicus*, hereafter referred to as *rat*) is one of the most abundant mammals with a nearly worldwide distribution (Galef, 2009; Puckett et al., 2016). Today, almost all wild rats live in close association with humans, leading to various forms of adverse interactions (Barnett, 2001). For example, rats are known to transmit diseases (Himsworth et al., 2013b), destroy stored food (Meerburg et al., 2009) and damage infrastructure by gnawing on wires or fundaments (Booy et al., 2017). Consequently, it has been estimated that rats cost only the US economy a minimum of two billion dollars each year (Pimentel et al., 2005). Furthermore, rats are predators that can be a threat to other species, especially when introduced to a new area (Moors et al., 1992). Thus, there has been a huge interest to control rat populations since mediaeval times (e.g., Barnett, 2001).

Today, controlling rat populations is becoming ever more important since the human population grows with increasing speed and in 2030 it is expected that more than 75% of all humans will live in cities, which means that they most likely will live together with rats (Cohen, 2003). Therefore, interactions with rats are likely to increase in the future, if the rat population control remains that ineffective (Parsons et al., 2017). At the same time, population control must not induce unnecessary pain or suffering in rats. To reduce rat populations humanely and effectively, however, detailed knowledge on their individual behaviour and social interactions is needed (Himsworth et al., 2013b; Parsons et al., 2017).

Whereas wild rats are undesired in close proximity to humans, domesticated rats are more than welcome as pet rats in households, where approximately 100’000 lived in 2019 in the UK alone (PFMA, 2020), and as laboratory rats in scientific institutes, where more than 20 million rats are used worldwide for research every year (Baumans, 2004). The use of rats have led to key advances in various fields (Suckow et al., 2006). The first described scientific experiment using rats as model organism dates back to the 1850s (Philipeaux, 1856). Ever since, rats turned into one of the most important model organisms, resulting in over 350 different laboratory strains available today (http://www.rrrc.us/search/). In 2004, the full genome of one strain (BN/SsNHsd) was sequenced, which further increased the possibilities of research in the fields of biochemistry, genetics, genomics and physiology (Gibbs et al., 2004). Today, it has been estimated that worldwide approximately one study is published per hour that used rats as model organism (Berdoy, 2002).

The use of any non-human animal species in research raises ethical issues, concerning under which conditions humans are allowed to use and potentially harm them. Moreover, it has been pointed out that poor welfare can impact the quality of science and may hence be one factor of the reproducibility crisis in science (Garner, 2005). In order to provide optimal handling and care and identify factors inducing potential harm and pain, detailed knowledge on the rat’s social needs are essential (Bracke and Hopster, 2006; Keifer and Summers, 2016).

Given their omnipresence and close association with humans, it is surprising how little is known about the rats’ individual social behaviour under natural conditions. One reason for this shortcoming is that rats as nocturnal and sub-terrestrial animals are notoriously difficult to observe. Despite these challenges, there is the general assumption that wild rats are not being particularly prosocial towards their conspecifics (e.g., Barnett, 1957; Feng and Himsworth, 2014; Inglis et al., 1996). This stands in stark contrast to recent laboratory studies showing that rats are highly social animals that depend strongly on conspecific cooperative interactions and consequently show elaborate prosocial behaviours, such as freeing trapped partners and exchanging favours (Mogil, 2019; Schweinfurth, 2020; Wrighten and Hall, 2016).

**Figure 1.**
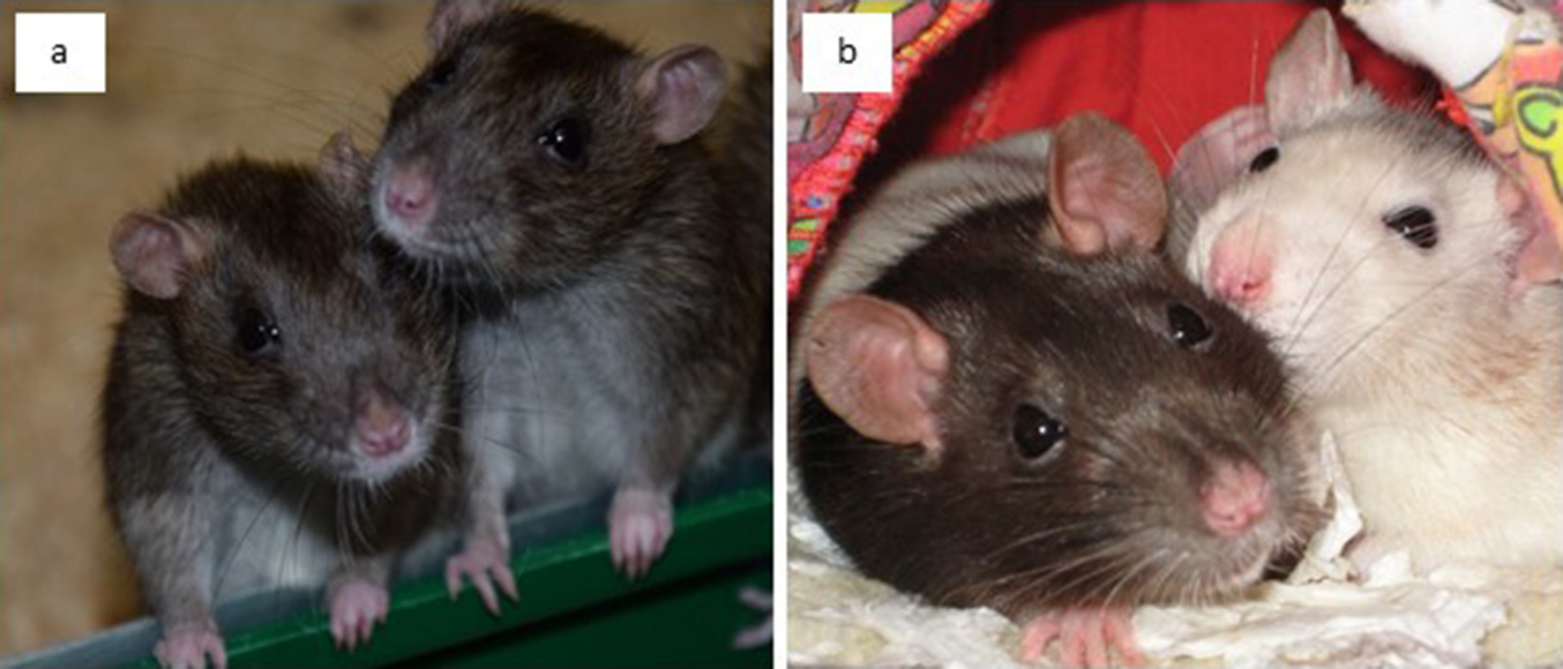
Wild and domesticated Norway rats. Wild rats (panel [**a**] depicts two female wild-derived rats) differ from domesticated rats (panel [**b**] shows two female domesticated rats) greatly in respect to their coat colour but less so in their social life, which is illustrated by domesticated rats showing the full behavioural repertoire of wild rats. Therefore, domesticated rats can be good representatives of wild rats and *vice versa*.

These different conclusions about the social behaviour of rats are surprising and require further investigations. One possibility is to compare the different lines of research on domesticated and wild rats. Since domestication of rats has started in the 19^th^ century (Lindsey and Baker, 2006), it is sensible to assume that selective breeding and adaptions in response to artificial environments occurred and hence wild rats differ from domesticated rats in their social behaviour. Furthermore, wild rats have the propensity to rapidly adapt to environmental changes (Johnson and Munshi-South, 2017). In accordance, direct comparisons between the behaviour of wild and domesticated rats show that, for instance, wild rats burrow less (Price, 1973), are more neophobic (Mitchell, 1976), learn more slowly (Boice, 1972) and are more aggressive towards conspecifics (de Boer et al., 2003). Consequently, wild rats dominate domesticated rats in direct interactions (Boreman and Price, 1972). However, the described differences between wild and domesticated rats are rather quantitative than qualitative (Figure 1). Indeed, domesticated rats show the full behavioural repertoire of wild rats (Boice, 1981; Boice, 1973). Consequently, domesticated rats have been shown to survive and reproduce under semi-natural conditions (Adams and Boice, 1983; Blanchard et al., 1988; Boice, 1977), even if predators were present (Berdoy, 2002). Thus, the behaviour of domesticated and wild rats can be representative for each other and hence the obtained knowledge on domesticated rats can inform studies on wild rats and *vice versa*. An integration of laboratory and field studies would therefore be highly valuable for all human interactions with this animal in general.

## Aim of this review

Although few other vertebrates have a similar impact on humans as the rat, we know surprisingly little about their individual social behaviour in nature. Furthermore, their social cognition has been largely ignored in developing management strategies for wild rats or lab protocols and welfare recommendations for laboratory rats. This review is an attempt to rectify this problem. In the first part of this review, I provide a summary of the social structure and the individual social behaviour of rats, as far as it is known. In the second part, I then review recent developments on the social skills of rats, mostly performed under controlled laboratory conditions. Finally, I elaborate on why an integration of both research lines would be desirable.

## Social behaviour of wild rats

Wild rats live almost exclusively in close contact with humans (Singleton et al., 2003). Still, knowledge of wild rats is almost exclusively based on a few early pioneering studies, which have rarely been pursued because formerly used methods are not considered ethical or safe enough anymore. Still, there are more recent studies that tested either wild-derived rats, that is laboratory-reared descendants of wild rats, or wild-caught rats in captivity. Here I aim at synthesising this body of literature, which has not been recently reviewed in the light of the rat’s social life, to make the information easily accessible to those working with rats. Given the new developments in the field of social cognition, a comprehensive review on the natural social behaviour is needed to align the disciplines and potentially inform each other. Throughout, I highlight the potential of more research on their social life for future research in various disciplines.

### The social structure

Wild rats live in large colonies, which, dependent upon food resources, may be composed of more than 150 individuals (Davis, 1953). Colonies are usually structured into subgroups, which might consist of pairs, harems with or without offspring, unisexual groups and/or single males and females (Calhoun, 1979; de Boer et al., 2016; Timmermans, 1978). Although wild rats frequently interact with other colony members, it is unclear how often they interact with members of different subgroups and how stable such subgroups are. Answers to such questions will greatly inform management strategies, by e.g. assessing which rats are likely to learn food avoidances or encounter contaminated faeces, and validate fundamental research, by e.g. investigating often made assumptions about the frequency of social interactions.

**Figure 2.**
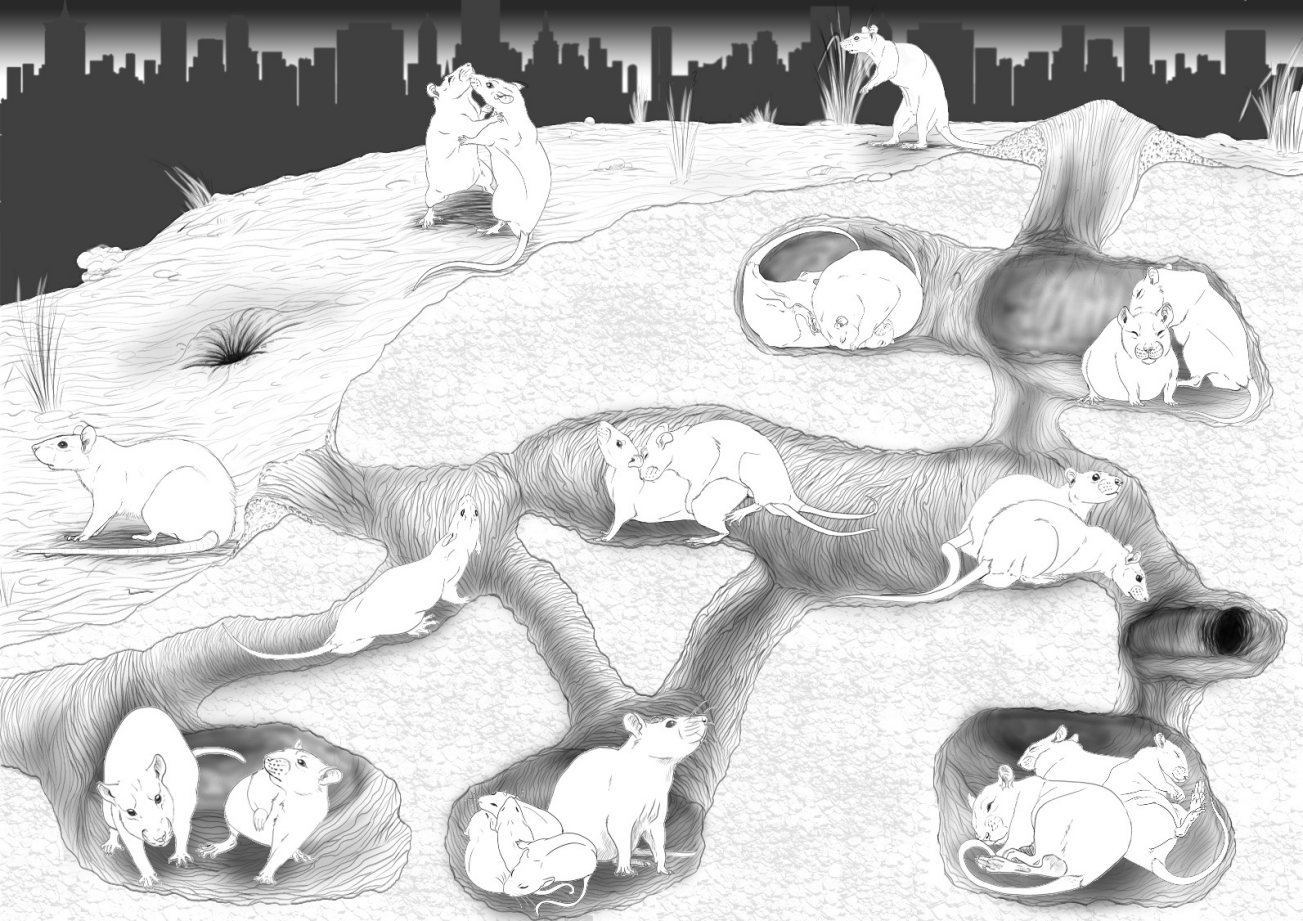
The social organisation of a rat colony. Rats are social animals that live in large colonies, consisting of sometimes more than 150 female and male individuals with overlapping generations. Rats of a colony establish an underground burrow system with shared channels and chambers. In these chambers, they commonly huddle together to keep warm and often sleep like this (right chamber at the bottom and left at the top). Females establish their nest in such chambers, where they give birth to up to eight pups (middle chamber at the bottom). Colony members reproduce. Males approach females that respond defensively with sidekicks, if they are not in oestrus (left chamber at the bottom). If she is receptive, several males will copulate with her (two rats in the middle). Rats establish a dominance hierarchy, which is more pronounced in males than females. When rats meet, they inspect each other, whereby subordinate individuals show a submissive posture, crawl under the other or avoid such contacts to prevent conflict. Most conflicts, however occur between rats of different colonies. Fights typically start with a threat posture, followed by fights that are interrupted by standing upright and boxing (two rats outside the burrow system). Most commonly, however, rats show amicable behaviour with colony members. For example, they spend time in close proximity to each other (left side, middle rats) or groom each other (right chamber at the top). Drawings by Michelle Gygax.

Wild rats jointly excavate a burrow system, which consists of tunnels and chambers that serve as shared nest sites as well as places for food storage (Telle, 1966). Since behavioural data is mostly lacking, it is unknown whether rats have task differentiation, e.g. digging, defending, or scanning the environment for predators. Outside their burrow, rats establish a trail system, which is marked by olfactory cues and serve as foraging paths as well as connections between sites (Telle, 1966). A schematic overview of the social structure of a rat colony is provided in Figure 2.

### Differential relationships

The social relationships between colony members can be highly variable. Studies on wild rats under semi-natural conditions showed that while mothers form strong bonds with their infants until these are around two months old, there is no evidence that adult wild rats form stable pair bonds (e.g., Barnett, 1963; Calhoun, 1979). In line with this, wild-derived females also do not form selective social bonds in captivity (Schweinfurth et al., 2017a). The lack of pair and social bonds is intriguing and stands in contrast to many other social mammals (Seyfarth and Cheney, 2012). Why rats show many social behaviours (see ‘Social cognition in rats’), but do not integrate social information into bonds deserves further investigation in order to elucidate their social activities with implications for housing recommendations.

What seems more important than bonds is an organisation based on dominance hierarchy. Probably based on their oestrus cycle, female rats form rather loose hierarchies under captive (domesticated and wild rats: Ziporyn and McClintock, 1991; wild-derived: Schweinfurth et al., 2017a) and semi-natural conditions (Calhoun, 1979). Under the latter conditions, males form more pronounced, stable and near-linear hierarchies based on fighting abilities (Berdoy et al., 1995). Three main male types have been identified (Barnett, 1963): Alpha males are the largest individuals in the colony, move freely and initiate attacks on intruders. Beta males shy away from alpha males but can gain weight. Lowest ranking omega males generally lose weight and do not reproduce. Therefore, omega males often disperse from their natal colony. The dominance hierarchies in rats are characterised and maintained by the exchange of various social behaviours (summarised in an ethogram, Table 1).

**Table 1.** Ethogram of individual social behaviours in rats. Rats show a range of social behaviour, that is behaviours that are directly related to conspecifics, which can be split into socio-positive and socio-negative contexts. The ethogram is restricted to wild rats under natural or semi-natural conditions.

Category	Behaviour	Sex	Description	Reference
Socio-positive	Allogrooming	Females and males	One individual gently nibbles or licks the fur of a conspecific, sometimes with the aid of its forepaws. All body parts of the partner may be cleaned including the tail.	Barnett, 1963, p. 77
	Huddling	Females and males	Rats lie together with direct body contact, sometimes sleeping.	Barnett and Spencer, 1951; Barnett, 1963
	Inspecting anogenital region	Females and males	One individual sniffs or licks the anogenital region of a conspecific, probably used in the context of recognition.	Barnett, 1963, p. 64
	Nosing	Females and males	One individual gently pushes another’s flank or neck with its nose.	Barnett, 1963, p. 77
	Nose-touching	Females and males	Two individuals approach each other until their noses come into contact. This possibly serves recognition and may result in socio-positive or negative behaviours.	Calhoun, 1979, p. 179
	Oral inspection	Females and males	One individual sniffs at a conspecific’s mouth. This is most common between mothers and their offspring, but takes place between adults, too.	Calhoun, 1979, p. 149
	Pioneering	Females and males	One individual leaves the burrow vigilantly and observes the surroundings for several minutes. Only then will other colony members appear from the burrow.	Barnett and Spencer, 1951; Telle, 1966
	Play fighting	Females and males	One individual attacks the nape of its opponent, which the latter tries to defend. Play fights take place only during adolescence.	Ewer, 1971; Calhoun, 1979, p. 180
	Recognition sniffing	Females and males	One individual shows enhanced sniffing at colony members and (potentially marked) objects, especially if a stranger entered its territory.	Barnett, 1967
	Scent marking	Females and males	One individual rubs the flanks or presses the anogenital region on a surface, sometimes leaving urine droplets on the surface.	Landete-Castillejos, 1997
	Sharing food	Females and males	An individual tolerates a conspecific in its close proximity, sometimes even touching each other, while feeding from the same food resource. Alternatively, one individual drops small food items that can be taken by another. Further, residues in the face or on the paws of an individual can be licked off by another.	Barnett and Spencer, 1951; Barnett, 1963, p. 36; Calhoun, 1979, p. 101
	Submissive posture	Females and males	One individual lies on its side with eyes half-closed. This posture is used to ‘greet’ more dominant individuals to prevent fights. Sometimes this posture is combined with ‘crawling under’ (see below).	Barnett, 1967
Socio-negative	Aggressive grooming	Mostly males	One individual pins down a conspecific forcefully while allogrooming it. This is often accompanied by squeaks and run-away attempts of the groomed partner.	Barnett, 2001, p. 131
	Avoiding	Females and males	One individual changes its route upon detecting another rat.	Calhoun, 1979, p. 179
	Boxing	Mostly males	Bouts of fights are typically intermitted by standing upright to box. While boxing, they hit and scratch each other’s face, which is accompanied with raised hair and ears pointing forward.	Barnett, 1963
	Chasing	Females and males	One individual runs after a second. This usually precedes fights but can also take place afterwards.	Calhoun, 1979, p. 181
	Crawling under/walking over	Mostly males	One rat crawls under, that is typically the subordinate, or walks over a conspecific, that is typically the more dominant.	Barnett, 1963
	Direct approach	Mostly males	An individual approaches an opponent to attack, often accompanied with urination and defecation and raised hair. Sometimes the individual shows tooth chattering while approaching.	Barnett, 1963
	Fighting	Mostly males	Two rats tumble, roll over the ground while holding, kicking and punching each other.	Barnett, 1963; Calhoun, 1979
	Leaping and biting	Mostly males	The attacker jumps towards the opponent with extended forelimbs and tries to bite usually its ears, limb or tail. Bites are typically very quick.	Barnett, 1963
	Passage guarding	Probably only males	One more dominant individual stands in a passage and therefore blocks the way of a second. The opponent typically waits until the first moves away or takes a detour.	Calhoun, 1979, p. 187
	Pushing away	Females and males	One individual pushes another with its forepaws or flank to move a conspecific from its current position. Sometimes pushes are accompanied with kicks or swinging the body towards the opponent.	Calhoun, 1979, p. 101
	Tail quivering	Females and males	One individual quivers its tail, which might be shown in various situations like during ‘crawling under’ or shortly before copulation.	Steininger, 1950
	Threat posture	Mostly males	An attacker adopts a posture where the back is maximally arched, all limbs are extended, and the flank is turned to its opponent.	Barnett, 1963
	Tooth-chattering	Females and males	One individual chatters with its teeth while staying immobile, most common after detecting an opponent.	Barnett, 1963

### Agonistic behaviours

Studies on wild rats under semi-natural conditions showed that overt aggression between colony members occurs infrequently (Barnett, 1963). Instead, most agonistic behaviours are directed against intruders from outside the colony (Blanchard et al., 1988). This might explain the comparatively low dispersal rates in wild rats (Gardner-Santana et al., 2009; Puckett et al., 2016). In general, males seem to be less socially tolerant than females (Calhoun, 1979). Hence, solely males patrol and defend the territory boarders. Females, in contrast, seem to be less territorial, but defend their breeding chamber when lactating (Barnett, 1963). Although domesticated rats are known to kill conspecifics under limited space conditions in the laboratory (e.g., Blanchard et al., 1975), there is no evidence that rats kill each other under natural conditions, in which there is enough space for separation (Barnett, 2001). It is possible, however, that rats evict others from the colony, resulting in evicted rats being prevented from feeding and facing an increased risk of predation, which may ultimately lead to the death of the evictees (Barnett and Spencer, 1951).

Factors that increase conspecific aggression and therefore decrease social harmony are of particular interest for welfare recommendations and management strategies. Providing an adequate social environment with minimal aggression for animals in captivity is important and challenging likewise. The same conditions should be avoided where they are undesired to prevent rat populations to grow. Although domesticated rats show lower levels of aggression compared to wild rats, they show the same repertoire of aggressive behaviours (Plyusnina et al., 2011). Hence, findings from wild and domesticated rats can reciprocally inform each other to understand how, for instance, group stability, partner choice, population density and physical environment (e.g., nesting possibilities and food distribution) can influence aggressive competition and social stress, which can be incorporated in welfare and management recommendations.

### Grooming and other affiliative behaviours

Rats engage in affiliative behaviours in various situations. A frequent affiliative behaviour in wild rats is allogrooming, where one individual licks or nibbles the fur of a conspecific (Barnett, 1963). In wild-derived female rats such allogrooming is directed preferably to spots that are difficult to reach for the groomed individual, such as the face or neck (Schweinfurth et al., 2017b). Wild rats experience such grooming already early in life, as it is directed from mothers towards infants. Later, infants transfer this behaviour to other colony members (Calhoun, 1979). Comprehensive data on the occurrence of this probably most important social behaviour in the wild is missing since it usually takes place invisibly underground. Hence, it is not known how much time wild rats spend allogrooming, in which situations they show allogrooming, and whether there are additional functions to hygienic reasons, as shown in primates (Dunbar, 1991). Such information has important implications for management protocols, because it allows predictions about the spread of diseases. It is also important for welfare recommendations, as it allows meaningful comparisons between the social behaviour of captive and free-living animals, which is called behavioural integrity.

Other affiliative or beneficial behaviours include food sharing, huddling and pioneering. As wild rats share the same feeding sites, some colony members share food with others either by allowing them to take food (Barnett, 1963; Galef et al., 2001) or by letting them lick off residues from their fur (Barnett, 1956). Huddling, where rats lie together piled in a heap on top of each other, is beneficial for thermoregulation and thus, common amongst altricial infants (domesticated rats: Alberts, 2006; Sokoloff and Blumberg, 2001; wild rats: Calhoun, 1979). Moreover, adult rats huddle together, even under warm conditions and when there is ample opportunity to separate, which suggests some social function beyond thermoregulation. When pioneering, one rat vigilantly leaves the burrow and observes the surroundings for several minutes. Only then will other colony members appear from the burrow as well (Barnett and Spencer, 1951; Telle, 1966). This behaviour is likely to be beneficial for colony members that stay in the burrow until the pioneer has scanned the environment for potential dangers.

### Play behaviour

Another socio-positive behaviour, which is mostly shown by young rats, is social play. Generally, social play behaviour is common among young animals as being part of the process of learning adult behaviour (Bekoff and Byers, 1988). Although wild rats have been reported to play with each other (Calhoun, 1979, p.180; Ewer, 1971), most evidence of social play is based on work on domesticated rats (reviewed in: Himmler et al., 2016). Domesticated rats engage in play fights, so-called rough-and-tumble play, when they are about 17 days old and retain this behaviour until they reach sexual maturity (Thor and Holloway, 1984). During play fights, young domesticated and wild-derived rats wrestle, box and kick each other in order to turn the opponent on the back and attack the nape (Himmler et al., 2013). The same study found that wild-derived rats show less social play compared to domesticated rats and their play involves less body contact. Domesticated rats emit 50 kHz calls during such play (Burgdorf et al., 2008), which are associated with positive emotions (Burgdorf and Panksepp, 2001) and hence point towards rewarding effects of social play. Studies on the neurobiology of this naturally rewarding behaviour can inform, for instance, research on human reward systems and psychiatric disorders (Trezza et al., 2010). Given that rats are a better model organism for such disorders than mice and effort in developing treatments is increasing, research in rats is predicted to increase in the future likewise (Abbott, 2009). To differentiate abnormal from normal behaviour and to establish a meaningful model, comparative studies under natural conditions are crucial.

### Reproductive behaviour

Wild rats reach sexual maturity at an age of two to three months (Clark and Price, 1981). Both sexes are highly promiscuous, and males are usually not able to monopolise single females due to the presence of many other males in the colony (MacDonald et al., 1999). Furthermore, compared to other mammals, wild females have a short oestrus cycle of one to two weeks (McClintock and Adler, 1978), which is further reduced to only four days in domesticated rats (Marcondes et al., 2002). Like other domesticated animals, domesticated males have been selected for increased fecundity and thus show also enlarged testes and reach maturity earlier than wild rats (Clark and Price, 1981; Setchell, 1992).

There is not much evidence for mate choice in wild rats. When living in large groups, several males follow the receptive female during their short receptive period before copulation (Berdoy et al., 1995). Most of the males successfully copulate with the female repeatedly (MacDonald et al., 1999; Steininger, 1950). After ejaculation, domesticated male rats in the laboratory insert a vaginal plug, which dissolves after 12 hr (Austin and Dewsbury, 1986), but so far there is no evidence for these plugs in wild rats.

Clearly, the rapid breeding of rats is one of the reasons for their success. Detailed knowledge on their reproductive behaviour in the wild, for example factors that promote or supress it, is needed to either prevent reproduction in the wild or promote reproduction in the laboratory. For instance, pregnant laboratory females resorb their embryos, if they smell cat urine in their nest during the first third of gestation (Voznessenskaya et al., 2002). This could be a non-invasive tool to discourage and prevent wild rats from nesting and breeding. Furthermore, contraception and contragestion as management tools have been recently revisited mainly because they are non-lethal (Miller and Fagerstone, 2000; Witmer et al., 2017). Importantly, mathematical models showed that such fertility control methods can be as effective as more traditional methods, such as baiting and trapping (Zhang, 2000). Still, reproduction in the wild is understood only very little.

### Mother-infant behaviour

Approximately three weeks after copulation, females give birth to a litter of four to eight pups in wild rats and eight to sixteen pups in domesticated rats (Calhoun, 1979; Clark and Price, 1981). Whereas domesticated rats breed communally under laboratory conditions (e.g., Mennella et al., 2014), this has not been confirmed under natural conditions (Barnett, 1963; Calhoun, 1979; Telle, 1966). Here, pups are born in a private underground chamber of the communal burrow (Davis, 1953). Males show no paternal care, whereas females nurse the altricial offspring until weaning at approximately 40 days (Barnett, 1963). Thereafter, the offspring stays in close association with their mother for up to two months (Calhoun, 1979), which stands in contrast to the common practice of breeders to separate offspring from their mother at the age of three weeks.

Maternal behaviour has profound long-term effects on offspring through changing their epigenome, which has been extensively studied in laboratory rats (reviewed in Champagne, 2008; Champagne and Curley, 2009; Liu et al., 2000; Weaver et al., 2004; Zhang et al., 2010). For instance, offspring of highly attentive mothers, which intensively lick and groom their offspring, show low plasma corticosterone levels, modest stress responses, better spatial learning, increased memory and become attentive mothers later in life, too. These maternal effects can affect biomedical research outcomes. If drugs are developed using rats that were raised by less attentive mothers or were separated from their mothers for even short periods, the sample will show significant behavioural and neurological changes (e.g., Champagne, 2008). Resulting drugs might only be effective and transferrable to humans that have also had faced considerable stress. Furthermore, elevated stress levels raise welfare issues in these animals. To establish what good maternal care means and associated variation, comparisons with wild rats in their natural habitat are indispensable.

## Social cognition of rats

Groups of wild rats are socially complex as they live in long-term groups of multiple generations, which allows for repeated interactions with differently familiar and related individuals (see Scheiber et al., 2017 for the characteristics of social complexity). Their complex social system implies that rats need certain cognitive adaptations to deal with challenges like mate or food competition that can be overcome, for instance, by cooperation or social learning (Byrne and Whiten, 1988). Yet, the social system of rats does not allow general conclusions about the underlying cognitive mechanisms of their behaviours. In recent years, there has been a considerable interest in understanding cognitive and emotional mechanisms of (mostly domesticated) rats when interacting with conspecifics. However, this recent development has not yet been aligned with the rat’s natural social environment. Hence in the second part, I elucidate the socio-cognitive skills of rats, which have mostly been studied under controlled laboratory conditions, to highlight the rat’s high social and emotional abilities.

### Recognising others

Recognising others is an important skill for almost all social behaviours. Recognition might be based on learning cues of familiar individuals or differentiating between classes, e.g. kin from non-kin. Both can be of relevance in mate choice, hierarchy formation or brood care (Penn and Frommen, 2010). Indeed, rats distinguish not only between kin and non-kin (domesticated rats: Hepper, 1987a; Zhang and Zhang, 2011; wild-derived rats: Schweinfurth and Taborsky, 2018a), but also between different degrees of relatedness on the level of cousins (domesticated rats: Hepper, 1987b). This ability is probably the result of prenatal imprinting, as rat pups prefer the amniotic fluid of their mother, even if they were born by Caesarean section (Hepper, 1987a). In addition, wild rats are able to discriminate colony members from intruders (Alberts and Galef, 1973). Remarkably, domesticated rats can truly differentiate between single conspecifics based on individually distinct odour cues after they were given the opportunity to learn such cues, instead of just using familiarity based heuristics (Gheusi et al., 1997; Hopp et al., 1985). This ‘true individual recognition’ is assumed to be cognitively challenging and has been demonstrated in only few other animal species (Tibbetts and Dale, 2007; Yorzinski, 2017).

### Emotion reading

Besides being able to identify others, domesticated rats are also capable of assessing the emotional state of other individuals, allowing them to predict future behaviour. This includes a differentiation of whether another individual is, for example, in a playful or aggressive mood. Rats show a large repertoire of facial expressions (Sotocinal et al., 2015), which are used to evaluate emotional states of conspecifics (Nakashima et al., 2015). Furthermore, they collect information of emotional states by odour cues from urine, which signal if another individual is stressed (Valenta and Rigby, 1968) or frustrated (Morrison and Ludvigson, 1970). These results highlight how sensitive rats are to the emotional states of conspecifics. The underlying mechanism of such emotion reading, however, is not yet clear. Rats may respond to certain emotions either by learned associations or alternatively by emotional contagion.

### Emotional contagion

Emotional contagion means sharing the emotional state with another individual. It is expressed by mimicking or being influenced by the response of other individuals (Preston and de Waal, 2002). This qualifies as a basic form of empathy without a cognitive understanding of another individual’s feelings. Indeed, it is assumed to represent the oldest form of empathy in evolutionary terms (de Waal, 2008), being present in many animal species, including humans. Emotional contagion serves an important adaptive value, that is saving time and energy by copying others instead of assessing and evaluating situations independently (Nakahashi and Ohtsuki, 2015). There is ample evidence that rats are capable of showing emotional contagion. When familiar with a particular stressor, domesticated rats stop moving, if realising that other individuals are experiencing this stressor (Atsak et al., 2011). Furthermore, if rats previously experienced electro-shocks for certain actions themselves, they stop pressing a self-rewarding lever, if they observe a partner being electro-shocked for their action (Church, 1959). Finally, rats learn to avoid situations that are harmful to others, even if they never experienced the harmful situation themselves (e.g., Knapska et al., 2010). Thereby it is not necessary for them to observe the behaviour of other rats because the smell of a stressed conspecific’s urine can elicit a stress response (Mackay-Sim and Laing, 1981).

### Helping behaviour

Rats not only use the knowledge about another individual’s emotional state for themselves, but also incorporate this knowledge to help conspecifics (summarised in Table 2). Rats have a high motivation to help each other, exemplified by their preference to cooperate, even if it is possible to achieve the same reward individually (Schuster and Perelberg, 2004). For instance, domesticated rats can coordinate their behaviour with conspecifics to avoid electric shocks (Daniel, 1942) or to gain rewards for themselves and their partner (Schuster, 2002). Such coordination for a mutual benefit is independent of sex (Schuster et al., 1993), strain (Schuster et al., 1988) or familiarity (Tan and Hackenberg, 2016) between the test animals. However, rats housed in isolation are not able to coordinate their behaviour with conspecifics (Swanson and Schuster, 1987), suggesting the social environment being necessary in developing social skills. When given the choice between delivering a reward to either themselves only or also to a partner at no cost, domesticated rats opt for the latter mutually-rewarding option (Hernandez-Lallement et al., 2016; Hernandez-Lallement et al., 2014; Márquez et al., 2015; Oberliessen et al., 2016). This consistent finding is surprising given that many other animals, including non-human primates, show only inconstant or limited evidence for helping conspecifics at no cost in similar prosocial choice tasks (reviewed in Marshall-Pescini et al., 2016).

**Table 2.** Overview of studies showing cooperative behaviour in rats. Rats are highly social animals that have been shown multiple times to cooperate, i.e. one individual benefits one or more other individuals (Sachs et al., 2004). Several mechanisms have been proposed to explain why they cooperate. Domesticated, wild and wild-derived rats of both sex were tested in a variety of tasks, involving various behaviours to measure their tendency to cooperate.

Proposed mechanism	Cooperative behaviour	Sex of test subjects	Origins of test subjects	Task	References
Assessing the other’s need in a helping task	Simultaneous nose-poking	Males	Domesticated (Sprague-Dawley)	Skinner box	Łopuch and Popik, 2011
	Entering one compartment, which leads to food rewards			T maze	Márquez et al., 2015
	Donating food by pulling it into the reach of a partner	Females	Wild-derived	Bar pulling task	Schneeberger et al., 2012; Schweinfurth and Taborsky, 2018b
Coordination (acting together)	Coordinating back and forth shuttling	Females and males	Domesticated (Sprague-Dawley)	T-maze	Daniel, 1942
					Swanson and Schuster, 1987
			Domesticated (S3, Sprague-Dawley and Wistar)		Schuster et al., 1993
			Domesticated (Sprague-Dawley and S3)		Schuster et al., 1988
		Males	Domesticated (Long Evans)		Tan and Hackenberg, 2016
Division of labour (sharing of tasks)	Tolerating thefts	Females and males	Domesticated (Wistar)	Diving for food	Colin and Desor, 1986; Krafft et al., 1994; Grasmuck and Desor, 2002
	Donating food by pushing down a lever	Males	Domesticated (Sprague-Dawley)	Skinner box	Littman et al., 1954
Empathy (ability to perceive and care for the emotional states of others) Or: social contact seeking	Freeing trapped partners by opening a door	Females and males	Domesticated (Wistar)	Partner trapped in container	Rice and Gainer, 1962
			Domesticated (Sprague-Dawley)		Ben-Ami Bartal et al., 2011
		Males			Ben-Ami Bartal et al., 2016
					Silberberg et al., 2014
		Females and males		Partner trapped in a pool	Sato et al., 2015; Schwartz et al., 2017
		Females	Domesticated (Sprague-Dawley and Long-Evans)	Partner trapped in container	Ben-Ami Bartal et al., 2014
Inequity aversion (preference of equal outcomes)	Entering one compartment, which leads to food rewards	Males	Domesticated (Long- Evans)	T-maze	Oberliessen et al., 2016
Prosociality (preference to provide benefits to others)	Entering one compartment, which leads to food rewards	Males	Domesticated (Long- Evans)	T-maze	Hernandez-Lallement et al., 2014, Hernandez-Lallement et al., 2016
			Domesticated (Sprague-Dawley)		Márquez et al., 2015
Reciprocity (conditional help based on previous received help)	Allogrooming	Females	Wild-derived	Direct interactions	Schweinfurth et al., 2017b
			Domesticated (Sprague-Dawley)		Yee et al., 2008
	Donating food by pulling it into the reach of a partner	Males	Wild-derived	Bar pulling task	Schweinfurth et al., 2019: Schweinfurth and Taborsky, 2018a
	Donating food by pushing down a lever		Domesticated (Long-Evans)	Skinner box	Li and Wood, 2017
	Entering one compartment, which leads to rewards		Domesticated (Sprague-Dawley)	T maze	Simones, 2007; Viana et al., 2010
	Donating food by pushing down a lever	Females and males	Domesticated (Long-Evans)	Skinner box	Li and Wood, 2017
	Donating food by pulling it into the reach of a partner	Females	Wild-derived	Bar pulling task	Rutte and Taborsky, 2007; Rutte and Taborsky, 2008; Dolivo and Taborsky, 2015a; Schweinfurth and Taborsky, 2016
Reciprocity between different commodities	Donating food by pulling und pushing it into the reach of a partner	Females	Wild-derived	Bar pulling and lever pressing task	Schwartz et al., 2017
	Allogrooming and donating food by pulling it into the reach of a partner			Direct interaction and bar pulling task	Stieger et al., 2017; Schweinfurth and Taborsky, 2018c
Warning	Alarm calling	Females and males	Domesticated (Long- Evans and Wistar)	Cat exposure	Blanchard and Blanchard, 1989; Blanchard et al., 1991
				Playback	Sales, 1991
		Males	Domesticated (Wistar)		Brudzynski and Chiu, 1995

Rats help conspecifics in several situations. For example, domesticated and wild-derived rats help each other by grooming spots that are difficult to reach by their own (Barnett, 1963; Schweinfurth et al., 2017b; Yee et al., 2008). Furthermore, rats warn colony members by producing alarm calls in the 20 kHz range (see 'Communication as a mean to mediate social behaviour'), to which conspecifics respond either by slowing down their movement (Brudzynski and Chiu, 1995; Sales, 1991) or by fleeing into their shelters (Blanchard and Blanchard, 1989). Domesticated rats in semi-natural enclosures are more likely to call in the presence of colony members, which suggests that the call is not a mere by-product of predator presence (Blanchard et al., 1991). However, a more recent laboratory study did not find that alarms calls were elicited more often in presence of conspecifics, which deserves further investigations of the mechanisms underlying alarm calls in rats (Wöhr and Schwarting, 2008).

In addition, domesticated rats have a high propensity to help trapped partners by opening a door of a restrainer (e.g., Ben-Ami Bartal et al., 2011; Rice and Gainer, 1962; Sato et al., 2015). This cannot be explained by mere curiosity to manipulate the door because rats preferably release familiar partners over strangers (Ben-Ami Bartal et al., 2014). In addition, the results are unlikely to be explained by seeking social contact (Schwartz et al., 2017; Silberberg et al., 2014) as rats free trapped partners even if social contact is prevented (Ben-Ami Bartal et al., 2011; Cox and Reichel, 2019) and rats only free partners when they are stressed (Sato et al., 2015). Furthermore, if treated with an anxiety-reducing drug, they release trapped partners less often, potentially because of reduced sympathy for the trapped partner (Ben-Ami Bartal et al., 2016). Taken together, rats adjust their help, which might be based on empathic concerns (Decety et al., 2016; Mogil, 2012; Panksepp, 2011).

Moreover, domesticated and wild rats have been shown to spontaneously share food with others, even if they have the chance of consuming the food on their own (Barnett, 1963; Colin and Desor, 1986; Grasmuck and Desor, 2002; Krafft et al., 1994; Littman et al., 1954). Food donations are fine-tuned to the partner’s need to receive help (Schneeberger et al., 2012), which may be communicated visually (Márquez et al., 2015), acoustically (Łopuch and Popik, 2011) or as a combination of several cues (Schweinfurth and Taborsky, 2018b). In summary, wild, wild-derived and domesticated rats help each other readily in various contexts, suggesting that, rats are highly cooperative at least in captive settings.

### Reciprocal interactions

When rats share food, they reciprocate help, that is they base their decision to provide food on previously received donations and pay back *quid pro quo*. More specifically, rats use at least two reciprocal rules. The rule ‘I help you because someone helped me’, that is generalised reciprocity, is followed by female, but not male wild-derived rats (Rutte and Taborsky, 2007; Schweinfurth et al., 2019). In addition, when interacting with the same partner repeatedly, rats use direct reciprocity, that is ‘I help you because you helped me’ (domesticated rats [Li and Wood, 2017; Simones, 2007; Viana et al., 2010; Wood et al., 2016], wild-derived rats [Dolivo and Taborsky, 2015a; Rutte and Taborsky, 2008; Schweinfurth et al., 2019; Schweinfurth and Taborsky, 2016; Schweinfurth and Taborsky, 2017; Schweinfurth and Taborsky, 2018c; Schweinfurth and Taborsky, 2018a; Schweinfurth and Taborsky, 2018c; Schweinfurth and Taborsky, 2020]). Thereby, they not only take into account whether the partner has helped them in the past, but also the quality of received help (Dolivo and Taborsky, 2015b) and the eagerness of the partner to provide help (Schneeberger et al., 2012).

While experimental laboratory studies on reciprocal food donations are inevitable to rule out alternative explanations, they have been criticised on the basis of their artificial design (McAuliffe and Thornton, 2015) and involved training (Zentall, 2015). To demonstrate their biological relevance, it is therefore necessary to show similar patterns also in naturally occurring behaviours that do not require any training. Two recent studies showed that wild-derived rats also exchange natural occurring allogrooming reciprocally (Schweinfurth et al., 2017b; Stieger et al., 2017). While allogrooming is a rather short affiliative behaviour, the reciprocal exchange has important life-long consequences, as rats that allogroom partners reciprocally live longer and develop fewer mammary tumours (Yee et al., 2008). Further, rats exchange food in an artificial setting against allogrooming as a natural behaviour (Schweinfurth and Taborsky, 2018c). This shows that artificial tasks can be suitable means to study reciprocity and that rats follow sophisticated rules when interacting with each other, which cannot be explained by training.

### Social learning and culture

Rats do not only benefit from conspecifics of which they receive help, but they also gain important information from others, for example about toxic diets (Steininger, 1950) or hunting techniques (Galef, 1982). They use this information even if it conflicts with their personal information (Galef and Whiskin, 2008; Galef and Whiskin, 2000). Learning to avoid certain diets has led to an arms race between the development of new poisons and the ability of wild rats to avoid these (Berdoy and Smith, 1993; Rost et al., 2009). When learning from others where to eat, wild and domesticated rats employ various methods (reviewed in Galef and Laland, 2005). First, they show local enhancement, that is visiting food locations where other rats were observed (Galef, 1971). Second, locations can be communicated by urine markings, which are used by others to locate suitable feeding sides (Galef and Heiber, 1976). Third, already before eating solid food, domesticated rats learn from their mother which food is palatable due to diet cues in the maternal milk (Galef and Sherry, 1973). Finally, domesticated adult rats can learn to copy preferences of other rats (Galef and Wigmore, 1983), which can be based on the breath of conspecifics (Galef et al., 1988). The so acquired preferences, learned over few encounters only, can last for at least one month (Galef and Whiskin, 2003).

Socially learned and transmitted information can result in group-specific behavioural patterns, shared by members of a community. Such patterns are commonly referred to as culture (Laland and Hoppitt, 2003). Indeed, wild rats show local differences in their hunting techniques that appear to fulfil the definition of culture. These local hunting traditions include raiding bird nests (Austin, 1948), hunting for birds up to the size of ducks (Steininger, 1950), fishing for small fish (Coleman et al., 1948) and diving to collect mussels (Gandolfi and Parisi, 1973).

### Social knowledge

Rats have been repeatedly shown to learn from conspecifics (see 'Social learning and culture'). They also observe others to infer information indirectly through transitive inference (Vasconcelos, 2008). This is particularly important in near-linear dominance relationships. For example, if an individual A is dominant over individual B and B over C, one can conclude that A might be dominant over C, even if this relationship was not directly observed. Domesticated rats can logically infer about hierarchical relations in artificial tests (Dusek and Eichenbaum, 1997; Roberts and Phelps, 1994): but see also Guez and Audley (2013), highlighting their socio-cognitive abilities.

### Communication as a mean to mediate social behaviour

Social interactions generally involve some form of communication, that is the intentional transfer of information between two individuals (Smith and Harper, 2003). Rats are known to use visual, acoustic and olfactory cues for communication (Whishaw and Kolb, 2004). As rats are active mainly at dusk and dawn (Telle, 1966), their visual system is poorly developed compared to diurnal mammals (Prusky et al., 2002). Accordingly, visual communication plays only a minor role during social interactions relative to other sensory modalities.

Rats communicate acoustically, both within and above (>20 kHz) the human frequency range. Audible for humans is the defeat cry, which is directed to con- and heterospecifics (Blanchard et al., 1986). Mostly, however, rats communicate within the ultrasonic range. This has been shown mainly in laboratory animals, without too much information on ultrasonic communication in wild rats. Three main ultrasonic calls have been described in domesticated rats (Brudzynski, 2009): an alarm call (frequency: 22 kHz, length: 300–3,000 ms), a social call (50 kHz, 20–80 ms) and an isolation call (40 kHz, 80–140 ms). While the former two types are emitted by adults and juveniles alike and occur in a variety of contexts, the latter are produced only by juvenile rats when they are separated from their litter or mother (Hofer, 1996). Social calls commonly are produced in a socio-positive context, that is during social play (Knutson et al., 1998), joint exploration (Brudzynski and Pniak, 2002), cooperation (Łopuch and Popik, 2011; Schweinfurth and Taborsky, 2018b) and during copulations (Barfield et al., 1979), as they may lead to approach behaviours by others (Wöhr and Schwarting, 2007). In contrast, alarm calls are usually associated with socio-negative contexts, that is when being threatened by predators (Blanchard et al., 1991), being exposed to fearful situations (Borta et al., 2006), showing submissive displays (Takahashi et al., 1983), but also during copulations (Barfield et al., 1979). Although fluctuation around 22 kHz, pitches of alarm calls differ across contexts, suggesting context specific modulations (Brudzynski, 2005).

Besides communicating vocally, rats also transfer information via odours (reviewed in Landete-Castillejos, 1997; Rennie et al., 2013). Such odour cues are used in a variety of contexts. First, domesticated rats actively scent mark objects or surfaces using urine or faeces by pressing their anogenital area or rubbing their flanks over the respective surface, possibly to signal dominance (Grant and Mackintosh, 1963). In wild rats, both sexes leave such marks probably for the same reasons (Telle, 1966). Furthermore, they mark the paths outside their burrow (Galef and Buckley, 1996) to optimise foraging efficiency and to increase protection from predators by using them as escape paths (Calhoun, 1962). In addition to urine, faeces are preferentially dropped at latrines or at places where rats of different colonies deposit urinary marks. This may suggest, that these areas serve as information centres (Frankova et al., 2015). Rats also possess glands across their body, whose odours transfer information of relatedness (Hepper, 1987a) and individual identity (Gheusi et al., 1997).

## Potential applications

Thus far, this review illustrated that the social system of rats is highly complex, both in the wild and in the laboratory. Rats form large socially structured colonies in the wild and exhibit various sophisticated social skills in the laboratory. Still, the inherent value of information on their social behaviour and their sophisticated social skills have not been acknowledged enough in welfare and management recommendations. Furthermore, current research on domesticated rats is often decoupled from information on their natural life and research on wild rats is decoupled of information on their social cognition, although such an integration could greatly inform each other. Hence in the third part, I discuss potential applications and future directions of such knowledge in different fields.

### Population management

Despite huge effort, controlling large rat populations has been mainly ineffective, especially in urban areas (Duron et al., 2017; Himsworth et al., 2013a). Possible reasons include bait/trap avoidance and recolonization of patches of which rodents were removed.

Trapping rats is notoriously difficult, exemplified by the fact that it took a team of several researchers 18 weeks to capture a single individual on a small island, although this animal was equipped with a radio collar (Russell et al., 2005). Reasons for this considerable trap-shyness are elusive. Here, behavioural studies could help identifying the reasons for their trap-shyness. For instance, some personality types might be more prone to be trapped than others (Biro and Dingemanse, 2009). Thus not all animals are equally well trappable, which will eventually lead to selection for trap-shyness, making future interventions more difficult. In addition, individuals might socially learn to avoid traps. Especially, their capacity for emotional contagion might be responsible for a strong negative association with traps (Knapska et al., 2010). While new traps have been designed and their efficiency been evaluated, the design should also consider welfare aspects. In 7% to 14% of all cases, traps do not kill the rats but injure them, causing ethical issues (Meerburg et al., 2008).

One of the most important aspects in controlling rats is to prevent them from recolonization a newly available habitat. Understanding the dispersal of rats and their attraction to certain habitats is necessary to successfully prevent them from recolonizing a cleared patch. However, thus far we know very little about how rats decide where, when and under which circumstances they start building a burrow. It should be noted that especially the social life of urban rats, although causing the most problems, is the least understood (Desvars-Larrive et al., 2018; Parsons et al., 2017). This is problematic because environmental factors strongly influence social and population dynamics in many animal species (e.g., Ebensperger et al., 2012; Groenewoud et al., 2016; Hill and Lee, 1998) and urbanization has profound effects on many species (Johnson and Munshi-South, 2017). Hence, the generalisability of social structure and immigration levels obtained from rats living in other habitats, e.g. farms, is currently unclear.

Finally, the killing of any animal causes an ethical conflict and information about the rat’s social behaviour might enable the development of new methods to pre-emptively repel them or hinder their reproduction, which is considered most effective by control professionals (Himsworth et al., 2013a). In addition, there is great demand for such strategies especially from organic farmers who aim to avoid rodenticides (Meerburg et al., 2004). Studying the rat’s mating behaviour might provide information on how to prevent or disrupt mating. Studying their nest-building behaviour might enable us to create unsuitable habitats targeted to rats. Furthermore, by increasing aggression between rats and decreasing social harmony, e.g. by blocking their communication, rats could be repelled.

### Research in Animal Behaviour and Psychology

Research on the rat’s behaviour and psychology has been mostly conducted under laboratory conditions, which comes with assets and drawbacks. While rigorous experiments under controlled conditions are indispensable when scrutinising possible mechanisms of behaviours, such tests may not be relevant in the rat’s natural world due to, for example, reduced attention or motivation. Thus, it is unclear whether observed patterns under laboratory conditions play an important role in the natural life of rats where they face various challenges, such as predation, hunger, competition or illness. That said, any skill discovered even in the absence of a seemingly biological relevance at present is of fundamental interest, as it needs to be addressed why they might possess this skill.

In order to produce generalizable scientific outcomes, various techniques can be applied (Bartels et al., 2018; Janmaat, 2019; Markman, 2018; Pearson et al., 2017). First, experiments in the laboratory can be designed to simulate naturally occurring tasks (Pearson et al., 2017; Schmuckler, 2001), such as by providing semi-natural conditions (Calhoun, 1979; Modlinska and Stryjek, 2016). Second, field studies may pose questions that have so far not been investigated and are thus a highly valuable resource to design laboratory tasks that investigate mechanisms of real-world problems (Campbell et al., 2009). Third, experimental research can be conducted in the field using novel techniques (see 'Future directions'). Thereby large samples can be studied in an ecologically relevant environment (Pritchard et al., 2016). Finally, interactions and collaborations between researchers studying rats in the laboratory and field will enhance external validity by combining expertise, of which this review is an attempt.

### Research in Biomedicine, Neuroscience and Pharmacology

Virtually all medical achievements have involved animal experiments at some stage, especially with rodents (Bateson et al., 2004). Choosing the best possible model organism for a test to measure what it claims to be measuring is the basis for the success of such studies (Greyer and Markou, 1995). Yet, test validity cannot be assessed without detailed information of rats in their natural environment and their cognitive and emotional skills (Olsson et al., 2003).

Every species evolved by adapting to a certain ecological niche, which has important consequences for biomedical research. If rats are tested in situations or with stimuli that do not resemble their ecological and social environment, they may not show an adaptive response, which might be different than that of humans in the same situation. Therefore, a direct comparison might be difficult. Even if naturally occurring stimuli are used, the frequency, duration and intensity of the stimuli should be closely matched to the natural situation to observe adaptive behaviour (Koolhaas et al., 2006). Studies of rats in their natural environment would help to inform biomedical research to design ecological relevant and translatable studies. This can be illustrated for research on stress responses. Rats have been traditionally given stressors that are arbitrary and artificial, like foot shocks, and their coping response is difficult to interpret (Koolhaas et al., 2006). However, stressors that elicit the highest plasma corticosterone responses are not artificial and involve social situations with conspecifics, like social defeat (Koolhaas et al., 2011). The natural history of rats can inform us about the predictability, duration and frequency of such events, different natural coping strategies and the natural variation therein.

Furthermore, some areas of biomedical research, for example neuroscience, enhance our understanding of mechanisms underlying abnormal behaviour. Often comparisons between normal and abnormal behaviours are necessary to identify and treat the latter. Such comparisons are only meaningful, if the control group is healthy and shows normal behaviour. Still, we mostly lack information about the rat’s ‘normal’ behaviour and individual variation of it, let alone information about naturally occurring abnormal deficits (Insel, 2007). In comparison to laboratory rats, wild rats live in a much more complex and variable social and physical environment. For example, wild rats build and live in large underground burrows where they interact with up to 30 times more group members than rats in captivity. Furthermore, group composition and size change regularly (Davis, 1953; MacDonald et al., 1999). Laboratory rats deprived of a complex environment show behavioural, neurobiological, physiological and psychological changes (reviewed in Fox et al., 2006; Girbovan and Plamondon, 2013; Lahvis, 2017; Laviola et al., 2008; Simpson and Kelly, 2011). This questions whether rats in control group show ‘normal’ behaviour and whether they are therefore a valid comparison to healthy humans. Thus, studies are needed that compare laboratory with wild rats to fully assess ‘normal’ behaviour and physiology. In addition, different captive settings should be explored where rats do not develop abnormal behaviour or physiology. This should include enriching their social life in captivity, for instance by housing them in large mixed-sex groups, which would at least partly resemble their natural social life.

Finally, restrictive standardisations has led to the testing of small subsamples, of which conclusions are hardly transferable to the whole human population (reviewed in Voelkl and Würbel, 2016). Test animals usually represent a homogenous group that is typically young, inbred and male. Moreover, test animals live in a restricted social and physical environment and share the same rearing and housing background. Findings obtained under such conditions are difficult to interpret because neither rats in other laboratories, nor in the wild, nor humans face similar restrictions (Kafkafi et al., 2018). Keeping rats under more diverse settings would mitigate this problem.

### Animal welfare

Scientific insight is only meaningful, if it is based on valid, reliable and repeatable experiments. All of these essential features of science can be reduced by poor welfare of the test animals (reviewed in Garner, 2005). In addition, there is increasing public concern about animal welfare in scientific studies (Baumans, 2004; Palmer and Sandøe, 2011; Webb et al., 2019). This is reflected, for instance, in animal right movements, animal protection organisations, constantly improved legislation and the development of animal ethics committees. Today, 75–100 million vertebrates are used in scientific studies per year, one third of which are rats (Baumans, 2004). Given this magnitude, the ethical treatment of rats in research is an important social issue.

Fundamental aims of good welfare include the animals’ well-being and positive emotions (Dawkins, 2006). Whereas most work has been done to improve the physical environment of laboratory animals (Patterson-Kane, 2004), enhancing their social environment has received less attention. Many studies have shown that isolated rats develop depression-like behaviours (e.g., Brenes and Fornaguera, 2008; Hurst et al., 1997). Their great social need is highlighted by the fact that rats prefer spending time with a conspecific over receiving heroin or methamphetamine, even if they are addicted to these substances (Venniro et al., 2018). They also put more effort in joining familiar conspecifics than inanimate objects or larger cages (Patterson-Kane et al., 2002). Finally, isolated rats showed marked differences in their behaviour, cognition, physiology and neurobiology that is likely to impact the translatability of results obtained on such animals (Arakawa, 2018; Mumtaz et al., 2018). All these studies present a strong case for housing rats socially.

However, social partners do not only provide comfort and entertainment, but can also cause stress and injuries. For instance, peer rejection during adolescence has been shown to have long-lasting effects in the brain and social behaviour of rats (Schneider et al., 2016). Therefore, it is crucial to house rats with appropriate partners, which has so far been largely neglected. Studying rats in the natural environment, in which they evolved, could give insights into individual interaction patterns as a function of space or disruptions of social dynamics and their resolution. Furthermore, we need to better understand causes of induced stress and aggression in wild rats to eliminate or reduce such factors in captivity. Severe physical attacks are risky and are thus usually avoided (Parker, 1974). However, early isolation from mothers might lead to poor social competencies (Bailey and Moore, 2018; Taborsky and Oliveira, 2012) and limited hiding opportunities might lead to escalations between individuals of similar ranks or unfitting personalities.

In addition to social housing, recent findings on the social skill set of rats should be incorporated in handling and experimenting protocols. Rats realise and respond to conspecifics that are stressed or in pain (reviewed in Chen, 2018). For example, rats that interact with a familiar partner in pain show afterwards stronger pain reaction themselves compared to rats that interact with an unfamiliar partner in pain (Li et al., 2014). Rats have been shown to possess mirror neurons that are active when experiencing pain and when observing a conspecific in pain (Carrillo et al., 2019). At the same time the presence of a partner can have buffering effects as rats seem to be less stressed when exposed to a stressor when in company of a cage mate (Kiyokawa et al., 2004) or when they can interact with a social partner shortly after a stressful event (Akyazi and Eraslan, 2014). In line with this finding, experiencing stressful situations together with a cage mate leads to a lack of fear conditioning towards such stressors (Kiyokawa et al., 2007), which might be important for experiments that are based on repeated treatments.

Chronically stressed laboratory animals are more likely to become aggressive, as well as physically and mentally sick (de Boer et al., 2017). This creates an unsafe environment for caregivers and can lower the generalisability of results. Knowledge on the cognitive and emotional skills of rats can be used to help rats to cope with stressful events. In addition, a detailed ethogram of wild rats can enable us to differentiate normal from abnormal behaviour, which is a very easy and non-invasive and way to assess the welfare of an individual and to make informed welfare recommendations.

Finally, a largely neglected welfare issue, but maybe an even more critical issue in terms of the affected numbers of rats, is the world-wide killing of enormous numbers of rats each year for population management purposes. Exact data on the number of killed rats is not available, besides an estimation of several million rats that were killed in the 90 s every year in the UK alone (reviewed in Mason and Littin, 2003). The currently available and legal control techniques can cause severe welfare issues. Poisoning, especially with anticoagulants, is the easiest, most effective and most common way of controlling rat populations (Mason and Littin, 2003). However, it is also the least humane way because it interferes with the rat’s ability to forage or escape predators, leaves sub-lethally poisoned individuals ill, affects non-target species (including children) and causes long-lasting discomfort and pain in poisoned and conscious individuals over an average period of 7.2 days (Mason and Littin, 2003; Meerburg et al., 2008). Furthermore, rat pups may die of dehydration or starvation, if their mother is killed and the distress through emotion reading and emotional contagion in colony members, which observe individuals in pain, is hard to assess. Systematic observations of rats when control procedures are applied in addition to knowledge about their social skills are needed to fully assess the impact of a particular procedure for developing recommendations. Furthermore, the development of contraceptive drugs, such as 4-vinylcyclohexene diepoxide (Witmer et al., 2017), seems a promising alternative because they are neither lethal nor toxic. Given that rats help others in need, read other’s emotions as well as feel with and potentially for each other, it is time to revisit and develop humane population control techniques that work pre-emptively.

### Future directions

Important questions about the social life of rats under natural conditions have not been addressed. While more detailed field studies would be highly valuable, it is notoriously difficult to study rats under natural conditions. Rats are small nocturnal animals that are visible only shortly during dusk and dawn. For the rest of the day, they live in underground burrow systems. However, newly developed technology, such as small cameras, camera traps, pheromone-based lures, microchips or high resolution GPS tracking software have become available and affordable (Parsons et al., 2016; Rutz et al., 2007; Strandburg-Peshkin et al., 2015). In addition, guidelines have been developed how urban rats, which might transmit diseases, can be studied (Desvars-Larrive et al., 2018; Parsons et al., 2017).

New research avenues to better understand the social behaviour of rats shall be taken by combining different disciplines. For example, given rats have an almost global distribution, this species would be ideal to study whether and how ecological factors influence their social behaviour, which can provide insights into the theory of social evolution. The gained knowledge on their behavioural ecology may inform comparative psychologists who study their social skills. By combining forces between pest control and behavioural studies, the gained information will shed lights on the individual behaviour of rats, which will inform humane population management projects. Three scientific disciplines, that is ethology, psychology and welfare have analysed the behaviour of rats for decades (e.g., Barnett, 1963; Hurst et al., 1997; Munn, 1950). Merging their theories, methods and findings would greatly complement each other. In turn, the results of these disciplines may affect the ethical judgment of some currently used population control techniques.
